# Cervical Necrotizing Fasciitis in an Uncontrolled Diabetic Male Patient: A Multimodal Management Approach

**DOI:** 10.7759/cureus.81177

**Published:** 2025-03-25

**Authors:** Srinivasa Swamy Bandaru, Paul Thomas Chirayil, Maher M Milhem, Rama M Almasri, Mahra Almazrouei

**Affiliations:** 1 General Surgery and Trauma, Saqr Hospital, Emirates Health Services, Ras Al Khaimah, ARE; 2 General Surgery, Ras Al Khaimah (RAK) Medical and Health Sciences University, Ras Al Khaimah, ARE; 3 Plastic and Reconstructive Surgery, Saqr Hospital, Emirates Health Services, Ras Al Khaimah, ARE; 4 General Surgery and Trauma, Ras Al Khaimah (RAK) Medical and Health Sciences University, Ras Al Khaimah, ARE; 5 College of Medicine, Ras Al Khaimah (RAK) Medical and Health Sciences University, Ras Al Khaimah, ARE

**Keywords:** cervical, debridement, idiopathic origin, multi-disciplinary, necrotizing fasciitis, skin grafting, wound negative pressure therapy

## Abstract

Cervical necrotizing fasciitis is a rare, life-threatening infection, often odontogenic. We report a case of idiopathic cervical necrotizing fasciitis in an uncontrolled diabetic patient, successfully managed through a multidisciplinary approach with good wound healing. A 54-year-old male with type 2 diabetes mellitus presented with a 10-day history of painful neck swelling and purulent discharge on the left side of the neck. Clinical examination revealed signs of necrotizing infection, confirmed by laboratory tests and contrast-enhanced computed tomography (CECT), which showed a large necrotic collection with gas formation extending to the supra-glottic region. Management included broad-spectrum antibiotics, insulin therapy, and urgent surgical debridement. Extensive necrosis involving the neck and laryngeal structures necessitated a second debridement on day seven, followed by negative pressure wound therapy. A split-thickness skin graft on day 14 led to complete healing, and the patient was discharged on day 21 with full recovery at the one-month follow-up. Cervical necrotizing fasciitis poses a high risk due to its proximity to vital structures and potential for mediastinal spread. Early diagnosis through imaging and clinical evaluation, along with aggressive surgical debridement and advanced wound care, is essential. This case underscores the importance of a multimodal strategy and optimal diabetes control in facilitating recovery and minimizing hospital stay. Idiopathic cervical necrotizing fasciitis, though rare, demands prompt diagnosis and intervention. An integrated approach, incorporating early imaging, repeated debridement, negative pressure therapy, and skin grafting, can significantly enhance patient outcomes. In this case, comprehensive management resulted in recovery within 21 days, shorter than the typical hospital stay for similar cases.

## Introduction

Cervical necrotizing fasciitis (CNF) is a rare form of necrotizing fasciitis (NF), representing less than 10% of all NF cases. It is less frequently observed compared to its occurrence in other anatomical areas [1]. The condition is predominantly of odontogenic origin [1,2]. Here, we report a case of CNF of idiopathic etiology in a patient with uncontrolled diabetes. The condition was effectively managed through a multidisciplinary approach encompassing otolaryngology, dentistry, general surgery, and plastic surgery. A comprehensive wound management strategy facilitated complete recovery and wound healing within three weeks of hospital admission. 

## Case presentation

**Figure 1:**
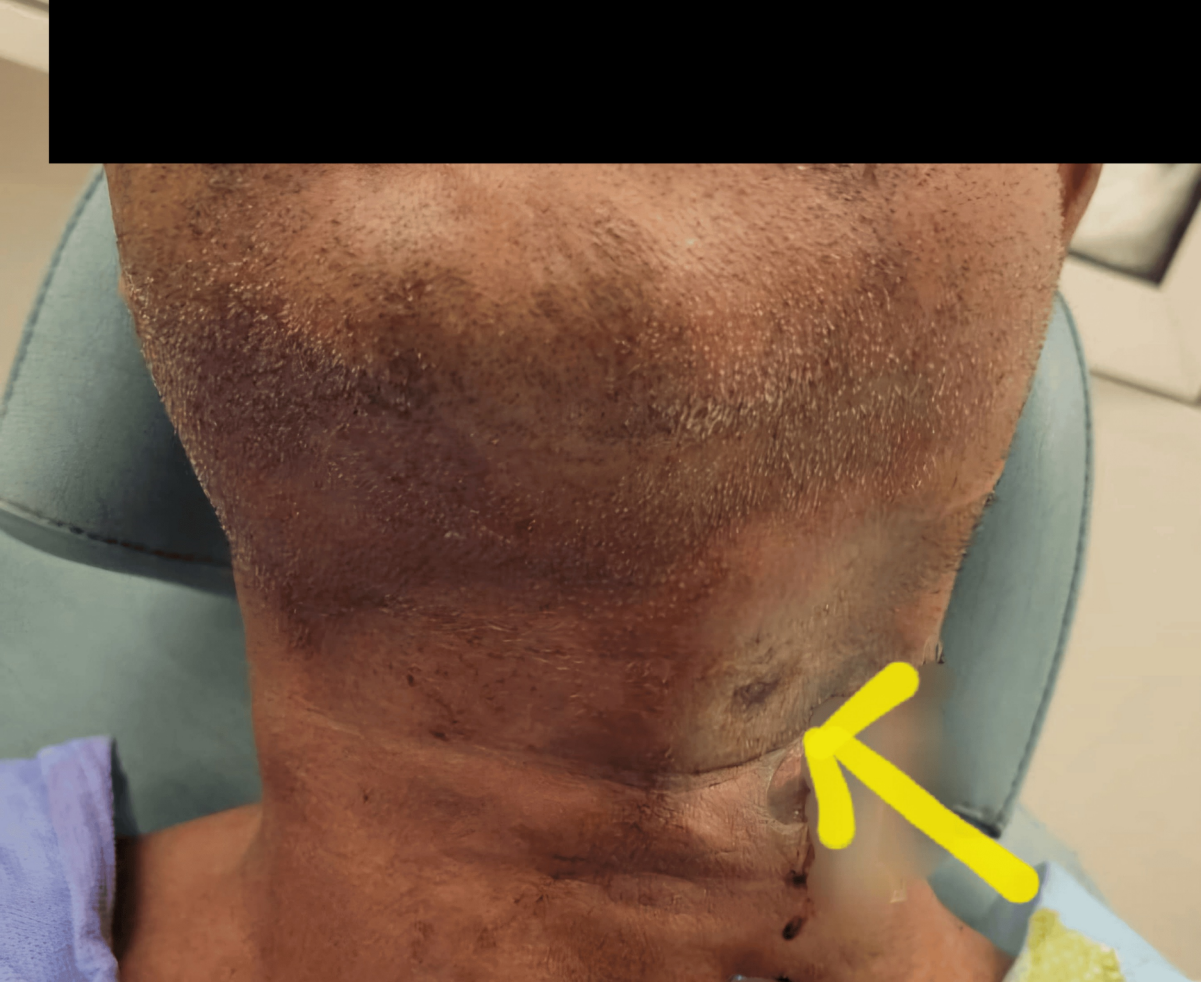
Preoperative image showing necrotizing infection (yellow arrow).

A 54-year-old male with a known history of type 2 diabetes mellitus with no past history of surgery was admitted with a 10-day history of painful swelling on the left side of the neck, accompanied by purulent discharge from the middle of the affected area. He had no associated respiratory symptoms. On initial evaluation, the patient was fully conscious and responsive, with a blood pressure of 155/110 mmHg, a pulse rate of 112 beats per minute, and normal respiratory rate and oxygen saturation. Otoscopic and nasal examinations were unremarkable, while the oropharynx exhibited copious secretions and a dental examination ruled out any dental pathology. Flexible laryngoscopy revealed medial displacement of the left lateral hypopharyngeal wall over the larynx. His body mass index (BMI) was recorded at 24. The patient had a history of poorly controlled diabetes and had not undergone any prior surgical procedures. Local examination revealed an abscess with purulent discharge and features of necrotizing infection on the left lateral neck, including erythema, crepitus, and tenderness upon palpation (Figure 1). 

**Figure 2:**
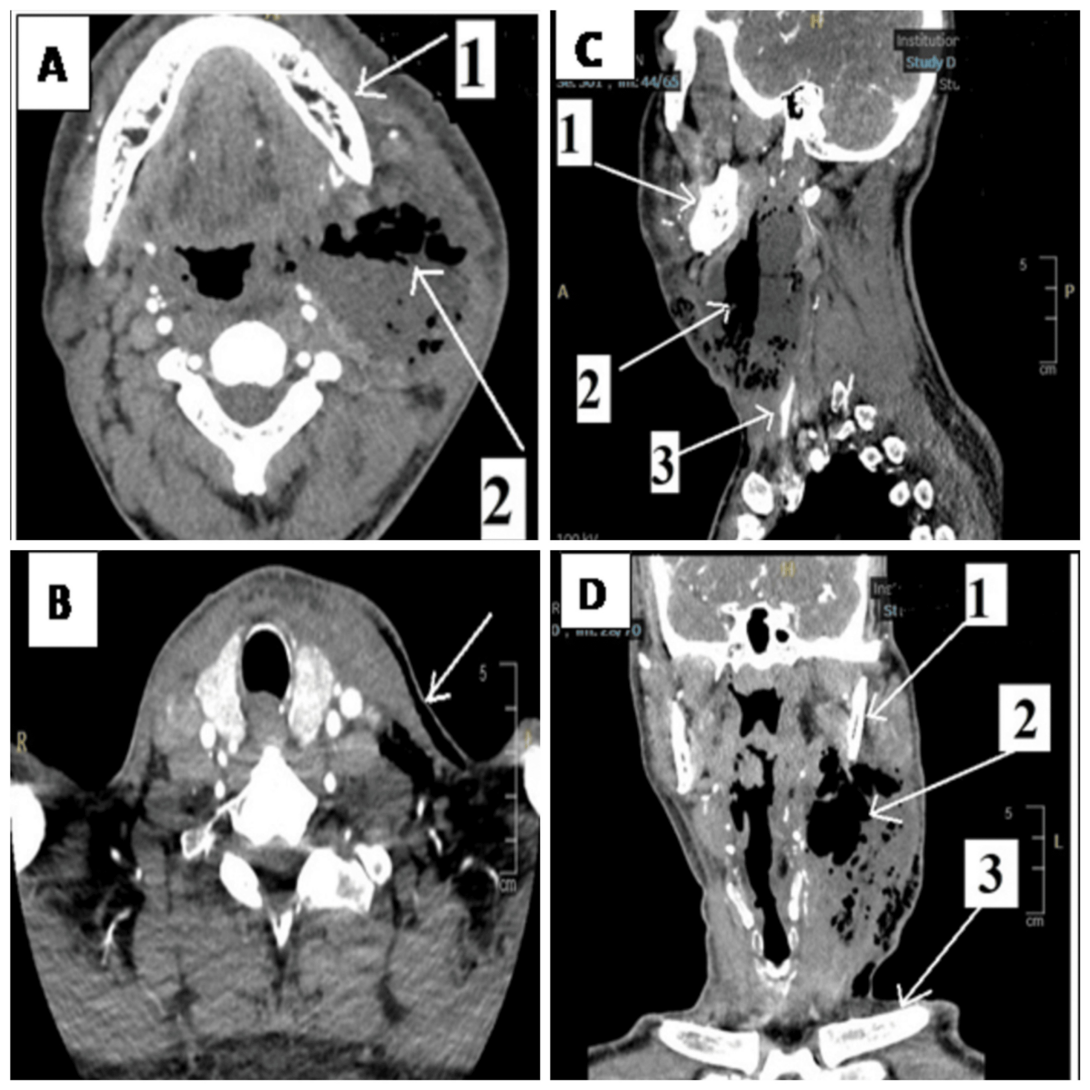
CT images showing the extent of necrotizing infection. (A) Axial view of the upper boundary: arrow 1 (mandible), arrow 2 (air and fluid). (B) Axial view of the lower boundary: arrow (subcutaneous air). (C) Sagittal and (D) coronal views: arrow 1 (mandible), arrow 2 (fluid with air shadow), and arrow 3 (clavicle).

Laboratory investigations at admission showed leukocytosis with a white blood cell (WBC) count of 20,110/mm³, hemoglobin (Hb) of 15 g/dL, and an elevated C-reactive protein (CRP) level of 274 mg/L. Platelet count, coagulation profile, and renal and hepatic function tests were within normal limits. The fasting blood glucose level was 16.8 mmol/L, and glycated hemoglobin (HbA1C) was 11.6%. Contrast-enhanced computed tomography (CECT) of the neck soft tissue revealed a large collection with marginal enhancement, measuring approximately 7 × 5.5 cm in axial dimensions (Figure 2A), located anterolateral to the carotid space on the left side of the neck. The collection contained significant amounts of air and pus (Figures 2A, 2B). The inflammatory process extended into the left pre-glottic space without crossing the midline and involved the supra-glottic larynx without evidence of sub-glottic extension (Figures 2C, 2D). 

Management

**Figure 3:**
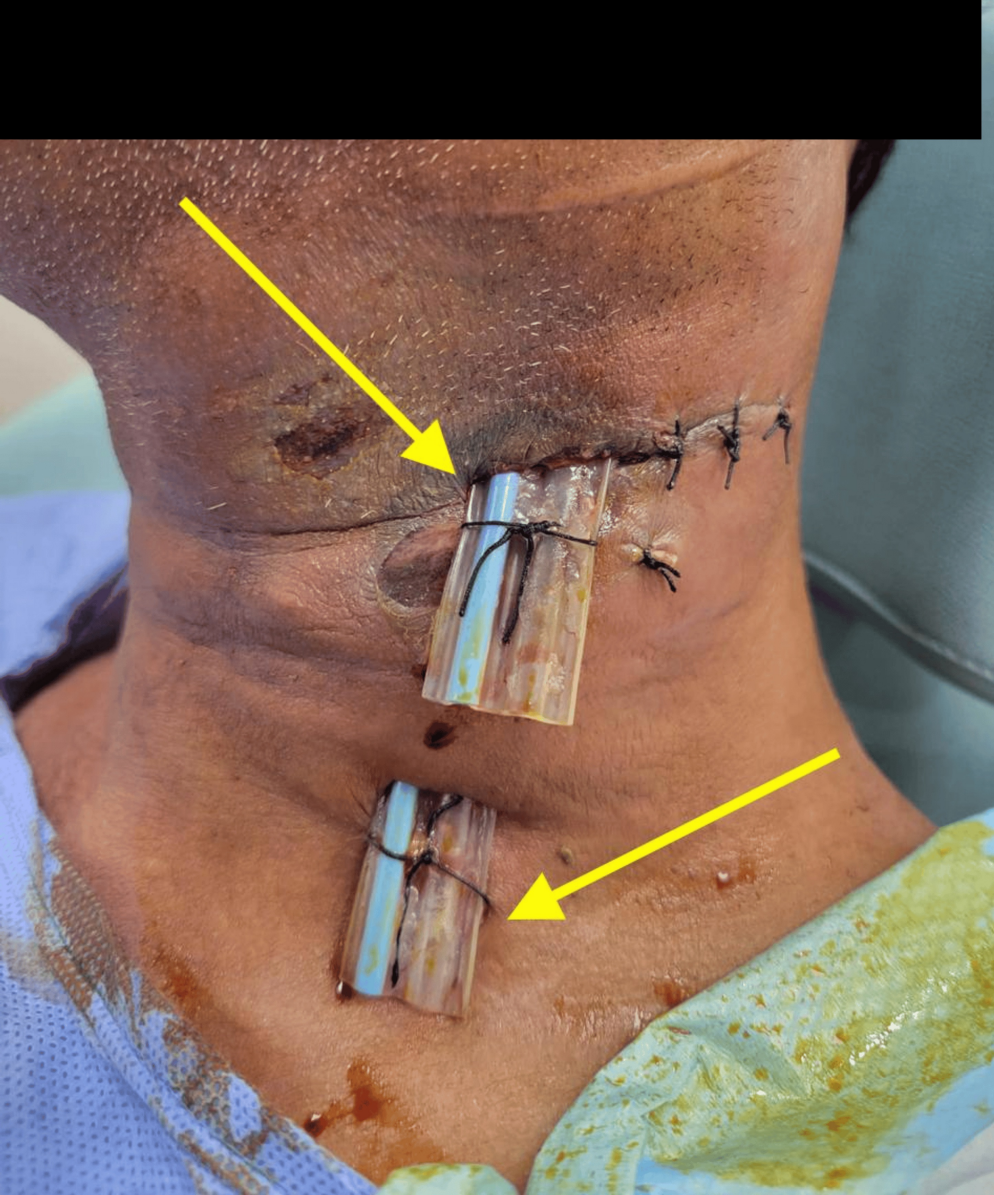
Image after first surgery with arrows pointing to corrugated drains.

A diagnosis of cervical necrotizing fasciitis was established. The patient was admitted and initiated on intravenous broad-spectrum antibiotics, insulin, and supportive therapy following an internal medicine consultation. Emergency surgical debridement and drainage of the necrotizing fasciitis were performed on the day of admission. Intra-operatively, extensive necrosis was observed on the left side of the neck, extending from the clavicle to the mandible and from the sternocleidomastoid muscle to the midline, not involving neurovascular, deep laryngeal structures and the airway with mostly viable overlying skin. A biopsy of the affected necrotizing infection was obtained, all necrotic tissue and pus were drained while preserving the overlying normal-appearing skin, and two corrugated drains were placed for drainage of collections (Figure 3). An endoscopic laryngeal assessment at the conclusion of the procedure confirmed a patent airway. Pus culture identified *Klebsiella pneumoniae* bacterial infection, which was sensitive to the empirically administered antibiotic regimen. Acid-fast bacilli culture was negative, and histopathological examination confirmed necrotizing fasciitis. 

**Figure 4:**
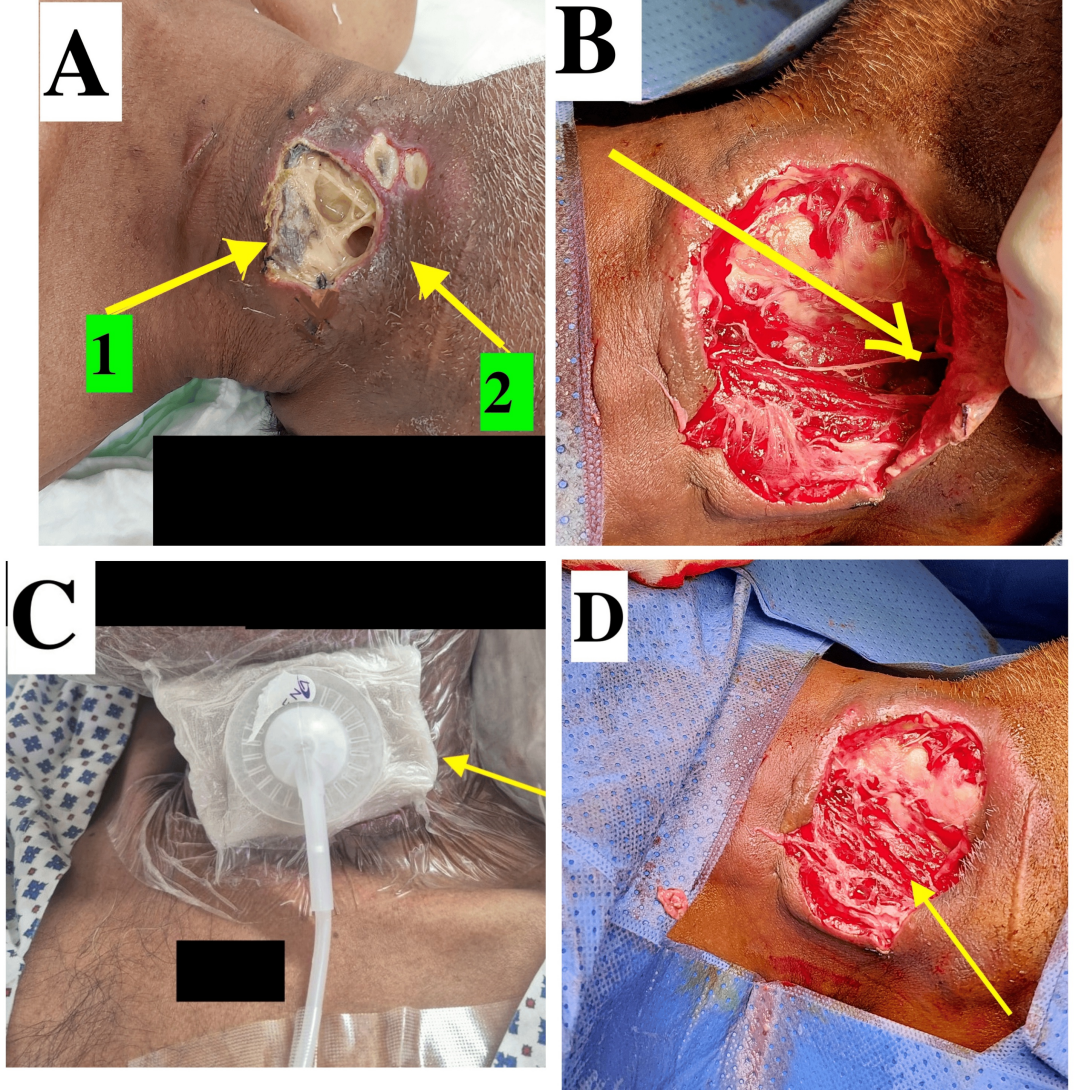
Intraoperative and postoperative images. (A) Day seven post-initial debridement, arrows (1) and (2) mark infection spread. (B) Post-second debridement wound status, an arrow pointing to the depth of the involved area. (C) With NPWT dressing. (D) Wound post-NPWT treatment. NPWT: negative pressure wound therapy.

**Figure 5:**
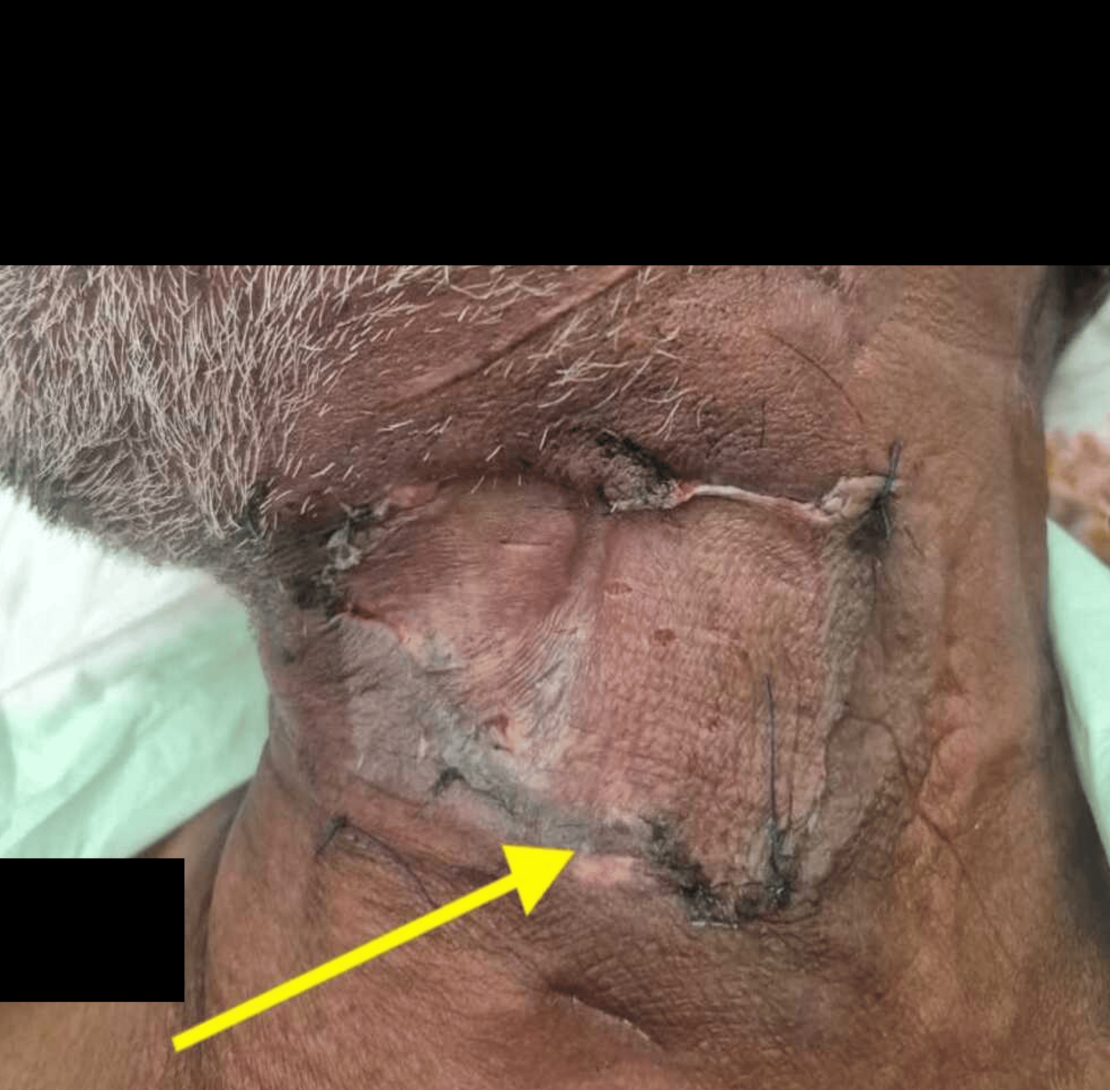
Post-skin graft follow-up image showing healed graft site with satisfactory integration (yellow arrow).

Postoperatively, the patient underwent regular wound dressings. However, due to the spreading necrosis of skin and fascia (Figure 4A), a second debridement was performed on postoperative day seven. This procedure resulted in a large raw wound on the lateral neck measuring 15 × 10 cm (Figure 4B). Following the second debridement, inflammatory markers, including WBC and CRP, normalized. Negative pressure wound therapy (NPWT) was initiated the next day of the second procedure and continued for six days (Figure 4C), leading to the development of healthy granulation tissue (Figure 4D). Seven days after the second debridement, a split-thickness skin graft was performed. Postoperatively, the graft was successfully taken, and the wound healed well (Figure 5). The patient was discharged on the 21st day of hospitalization. Complete wound healing was achieved within three weeks. At the one-month follow-up in the surgical outpatient clinic, the wound was fully healed.

## Discussion

Based on the origin of infection, CNF may be classified into odontogenic, non-odontogenic, and unknown origin (idiopathic) types, and based on the extent of spread, limited to neck type, and extended to upper chest type [1]. In a published meta-analysis by Gunaratne et al. [2], the odontogenic origin was found in 47% of cases, 35% were non-odontogenic (mainly throat), and the rest were unknown origin. In most cases, the etiological bacteria were primarily Streptococcus and Staphylococcus, with Gram-negative bacteria accounting for only 10% [2]. The case we reported had no identifiable infection focused on the mouth or ear, nose, and throat area, classifying it as of unknown origin. The organism identified was *Klebsiella pneumoniae*, a Gram-negative bacterium.

CNF presents clinically as a progressive spreading infection of the skin and subcutaneous tissue associated with signs of inflammation and the presence of crepitus with or without associated sepsis [3]; this distinguishes it from other conditions like cellulitis. A contrast-enhanced computerized tomography scan helps in the definitive diagnosis of the condition [4]. 

Necrotizing fasciitis in the neck area is potentially more dangerous than other common areas of the body, like lower limbs and perineal areas, due to its proximity to the vital structures like the larynx, trachea, vessels of the brain, and dangers of invasion of the infection into the mediastinum result in morbidity and reported mortality of 10-35% [5-7]. So, the early detection of this condition and prompt management in terms of appropriate debridement along with broad-spectrum antibiotics and controlling comorbidities are key to successful outcomes. 

The involvement of the neck area requires a multidisciplinary approach consisting of ear, nose, and throat surgery, general surgery, and plastic surgery [8,9]. Necrotizing fasciitis requires extensive debridement to control the spread [10]; it often results in the creation of big wounds, which might throw challenges in managing them [10,11]. Prompt management of the wound with available advancements in wound care and multimodal management like wound negative pressure treatment combined with proper wound dressings, like in our case, helps in faster granulation and reduction in the size of the wound and helps in early wound covered by a skin graft leading to faster recovery [11-14]. Emphasis should also be placed on controlling the medical comorbidities of the condition to get optimal results in a shorter time span. The mean hospital stays across several maintained analyses reported 27 days, whereas in our case, it was 21 days [2,10,14].

## Conclusions

Cervical necrotizing fasciitis of unknown origin is a rare condition compared to its occurrence in other parts of the body. Individuals with uncontrolled comorbidities, such as diabetes, are more vulnerable to developing this infection. Early recognition, supported by CT imaging, is crucial for timely diagnosis and intervention. Effective management requires a multidisciplinary approach, including thorough preoperative evaluation, identification of the infection source--whether odontogenic or non-odontogenic--through dental and ear, nose, and throat assessment, repeated surgical debridement, and advanced wound care techniques such as negative pressure therapy and skin grafting. Additionally, optimal management of underlying medical conditions plays a key role in accelerating wound healing, as demonstrated in our case, where recovery was achieved within 21 days. The wound management approach used in the reported case can also be effective for treating similar non-CNF wounds in the neck.
